# Severe conjunctivochalasis in association with classic type Ehlers-Danlos syndrome

**DOI:** 10.1186/1471-2415-12-47

**Published:** 2012-09-03

**Authors:** John K Whitaker, Philip Alexander, David YS Chau, Naing L Tint

**Affiliations:** 1 Division of Ophthalmology and Visual Sciences, Queen Medical Centre, University of Nottingham, Nottingham, NG7 2UH, UK; 2 Division of Ophthalmology and Visual Science, B Floor, Eye, ENT Centre, Queens Medical Centre, Nottingham, NG7 2UH, UK

**Keywords:** Conjunctivochalasis, Ehlers-Danlos syndrome, Kyphoscoliosis

## Abstract

**Background:**

Inferior conjunctivochalasis is common, but is rarely severe enough to require conjunctival excision. This report describes a patient with severe conjunctivochalasis who was subsequently diagnosed with Ehlers Danlos Syndrome, Classic Type.

**Case presentation:**

A patient suffering from foreign body sensation, frequent blinking and bilateral inferior conjunctivochalasis was referred and treated by topical ocular lubrication. However, no improvement was observed prompting potential excision of conjunctivochalasis. Following patient consultation and clinical diagnosis including hypermobile joints and skin elasticity, poor wound healing and wide scar morphology, Ehlers-Danlos syndrome was confirmed in the patient.

**Conclusion:**

This case highlights the need for direct patient questioning and provides the first reported association between conjunctiovochalasis and Ehlers-Danlos syndrome.

## Background

Ehlers-Danlos syndrome is a heterogeneous group of conditions characterised by skin hyperextensibility, atrophic scarring, joint hypermobility and generalized tissue fragility. The current classification includes six subtypes based on clinical, biochemical and molecular characteristics. However, examples of unclassified variants and 'overlap phenotypes' are becoming more common [1,2]. Kyphoscoliosis type (formerly type VI) and vascular type (formerly type IV) are associated with ophthalmic disease, though less commonly with the latter [3]. Known ophthalmic complications include ocular fragility with increased vulnerability to trauma, high myopia, retinal detachment and keratoconus. Epicanthic folds, microcornea, blue sclera, ectopia lentis and angioid streaks have also been associated, though less commonly [4,5].

Conjunctivochalasis can cause a spectrum of symptoms, ranging from dry eye, to disturbance of tear outflow, and exposure problems at the severe stage. It is frequently seen in the older age group as an elevation of the bulbar conjunctiva lying along the lateral or central lower lid margin and is often considered to be a senile degeneration with no reported systemic associations [6]. Studies of the clinical and histopathological characteristics of conjunctivochalasis, have suggested the aetiology to lie in a variety of local effects such as persistent trauma related to blinking, ultraviolet radiation or tear stasis. Possible systemic causes were not described [7].

We present a case of severe conjunctivochalasis in a middle-aged patient with Ehlers-Danlos syndrome.

## Case presentation

**Figure 1:**
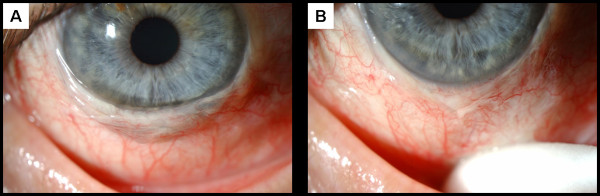
**(A) Severe conjunctivochalasis resulting in coverage of the inferior cornea. **(**B**) Conjunctiva displaced inferiorly using a cotton tipped applicator demonstrating the extent of the problem.

**Figure 2:**
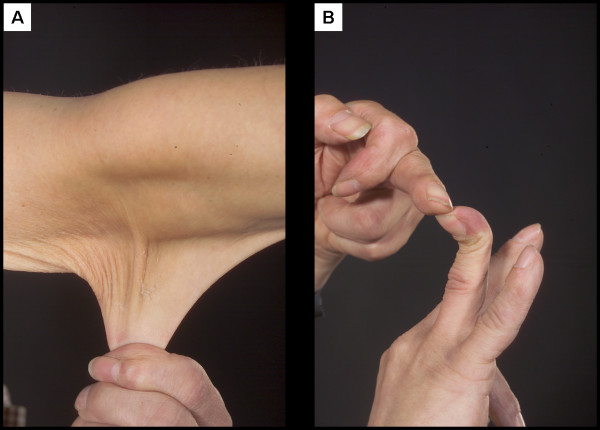
(A) Prominant hyperextensible skin (B) hypermobile joints.

A 55 year old male who was concerned about the appearance of his right eye over the past year was referred by his optometrist to the ophthalmology department. He reported a 12 month history of foreign body sensation causing frequent troublesome blinking. He had no symptoms suggestive of inflammatory ocular disease. He denied any other significant past medical history including atopic, allergic and skin disease at this stage. Ocular examination revealed bilateral inferior conjunctivochalasis, most notable at the right inferior corneal margin (Figure 1). There was no superior conjunctivochalasis. There was no evidence of meibomian gland disease or tear film instability. Fluorescein staining of the cornea did not reveal any punctate epithelial erosions. Topical ocular lubrication was commenced, but there was no symptomatic improvement. He was therefore offered excision of his right conjunctivochalasis under local anaesthesia. Informed consent was obtained prior to the surgery, at which time the procedure was discussed with him in lay language. In particular it was explained that the ‘excess skin covering the eye’ will be excised. This prompted the patient to volunteer the symptoms of joint hypermobility and skin elasticity (Figure 2). Further ophthalmic examination revealed no lens subluxation or blue sclera. The posterior segments were unremarkable and there were no angioid streaks.

Consultations were sought from Dermatologist and Clinical Geneticist. A diagnosis of Ehlers-Danlos syndrome (EDS), Classical Type (Type I/II in old nosology) was made based on the clinical findings of joint hypermobility, skin hyperextensibility, and wide scar morphology, which is indicative of tissue fragility and poor wound healing. Molecular testing, although available at the time, was declined by the patient. Cardiovascular assessment was unremarkable; in particular, there was no evidence of mitral valve prolapse on clinical examination or following echocardiography. No other members of his family were affected.

The patient’s symptoms improved significantly after the surgery. The conjunctival specimen was sent for histopathological analysis, which revealed evidence of marked damage to the lamina propria collagen, with mild regenerative atypia in the overlying epithelium. This is reported as a non-specific change that would not be directly linked to EDS or any other collagen disorder.

## Discussion

To our knowledge this is the first report of conjunctivochalasis in association with Ehlers-Danlos syndrome. It is possible that the combination of EDS and severe conjunctivochalasis in the same patient may be coincidence, but this seems unlikely. Severe conjunctivochalasis necessitating conjunctival surgery is rare in our clinical practice; EDS is also rare within our population. We hypothesise that is a true association, rather than a coincidence, especially given that the underlying abnormality in both the skin and the conjunctiva is related to collagen elasticity.

The potential mechanism for conjunctivochalasis to occur in this patient warrants some discussion. We hypothesise that the underlying collagen disorder either causes hyperelastic conjunctiva, or more likely predisposes to elastotic degeneration related to sunlight exposure. Ultraviolet radiation has previously been associated with conjunctivochalasis [7]. The superior conjunctiva is shielded by the brow and may therefore receive less sunlight exposure than the inferior conjunctiva; this could explain why inferior conjunctivochalasis is generally more common than superior conjunctivochalasis.

The diagnosis of EDS, classic type is established by family history, clinical examination and also using a standardised molecular test where appropriate [8]. Ophthalmic involvement has not been reported in this subtype. Over 50% of classic EDS patients have an identifiable mutation in genes encoding type V collagen, but quantitative and qualitative studies of type V collagen chains are usually not useful in confirming a diagnosis. Furthermore, a recent report has shown that mutations in the type V collagen genes may cause EDS phenotypes that differ from classic EDS [9].

## Conclusion

This case highlights the importance of direct questioning of patients presenting with conjunctivochalasis regarding signs and symptoms of Ehlers-Danlos syndrome, which is a potentially life threatening systemic disorder.

### Informed consent

Written consent was obtained from the patient for publication of this material. A copy of the consent is available for review.

## Competing interests

The authors have declared that no competing interests exist.

## Authors’ contributions

JKW: patient interaction and diagnosis, drafting of manuscript, final approval of manuscript, PA: patient interaction and diagnosis, final approval of manuscript, DYSC: patient diagnosis, final approval of manuscript, NLT: patient interaction and diagnosis, final approval of manuscript.

## Financial support

No financial support was received for this submission.

## Pre-publication history

The pre-publication history for this paper can be accessed here:

http://www.biomedcentral.com/1471-2415/12/47/prepub
